# Time series-based bibliometric analysis of a systematic review of multidisciplinary care for opioid dose reduction: exploring the origins of the North American opioid crisis

**DOI:** 10.1007/s11192-021-04154-z

**Published:** 2021-10-12

**Authors:** Abhimanyu Sud, Darren K. Cheng, Rahim Moineddin, Erin Zlahtic, Ross Upshur

**Affiliations:** 1 Department of Family and Community Medicine, Temerty Faculty of Medicine, University of Toronto, Toronto, ON Canada; 2 Bridgepoint Collaboratory for Research and Innovation, Lunenfeld-Tanenbaum Research Institute, Sinai Health, Toronto, ON Canada; 3 Institute of Health Policy, Management and Evaluation, University of Toronto, Toronto, ON Canada; 4 School of Kinesiology, Western University, London, ON Canada; 5 Dalla Lana School of Public Health, University of Toronto, Toronto, ON Canada

**Keywords:** Time series, Bibliometrics, Opioid, Multidisciplinary, Systematic review

## Abstract

Bibliometric analyses of systematic reviews offer unique opportunities to explore the character of specific scientific fields. In this time series-based analysis, dynamics of multidisciplinary care for chronic pain and opioid prescribing are analyzed over a forty-four year time span. Three distinct periods are identified, each defined by distinct research areas, as well as priorities regarding the use of opioids and the appropriate management of chronic pain. These scientometrically defined periods align with timelines identified previously by narrative historical accounts. Through cross-correlation with a mortality time series, a significant two-year lag between opioid overdose mortality and citation dynamics is identified between 2004 and 2019. This analysis demonstrates a bidirectional relationship between the scientific literature and the North American opioid overdose crisis, suggesting that the scientific literature is both reflective and generative of an important health and social phenomenon. A scientometric phenomenon of memory lapse, namely an overt and prolonged failure to cite older relevant literature, is identified using a metric of mean time to citation. It is proposed that this metric can be used to analyze changes in emerging literature and thus predict the nature of clinical and policy responses to the opioid crisis, and thus potentially to other health and social phenomena.

## Background

Multidisciplinary care (MDC) as an approach to managing chronic pain has been in development since at least the post-World War II period (Fordyce et al., 1968; Meldrum, 2003). This approach is based on a distinct conception of chronic pain as not only a biological or physical disease process, but instead as “biopsychosocial” (Engel, 1977). Namely, the origins and impacts of pain are often seen not only in bodily signs and symptoms, but also in terms of psychological effects such as depression, anxiety and stress as well as overall socialization and day to day functioning (Schatman, 2010). This holistic view of the chronic pain experience implicates treatments that attend to the various levels of impact and thus requires involvement from various professionals–from physicians to psychologists to physiotherapists to nurses, who have expertise in delivering these interventions. Often these treatments have been concatenated into interprofessional, multidisciplinary approaches to care–examples of which have been developed globally, initially led by the United States (US) (Bonica, 1990).

Multiple evaluations and systematic reviews, drawing from international experience and dating as far back as the early 1990s (Flor et al., 1992), have demonstrated effectiveness of MDC for people living with chronic pain. Despite this evidence of efficacy and also the high burden of complex chronic pain, there is persistent lack of access to MDC for chronic pain even in mature, high resource health systems such as those of the United Kingdom, Canada and the United States (Jeffery et al., 2011; Peng et al., 2007). Indications are that this access has been in steady decline over the last few decades (Choinière et al., 2020; Schatman, 2011). Instead through the 1990s and persisting until today, there has been a well-documented and dramatic rise in opioid analgesic prescribing for the management of chronic pain (Pain & Policy Studies Group 2016). This is despite opioids having had little to no evidence for long-term efficacy and persistent concerns around potential harms (Dowell et al., 2016; Krebs et al., 2018). Some have suggested that the decline of MDC access and the rise of opioids to treat chronic pain are directly related (Schatman, 2011).

Recently, as harms from prescribed opioids have continued to grow as part of what has been deemed an opioid crisis in North America, there has been resurgent interest in MDC as an alternative to opioid therapy and also specifically as potentially a mechanism through which to reduce harms from opioids (Ballantyne, 2007; Schatman, 2018). For example, in Ontario, Canada the Ministry of Health and Long-Term Care allocated C$17 million of annual funding to support the operation of 17 tertiary-level MDC pain programs across the province. Importantly, however, this funding was announced not as part of a provincial chronic pain strategy, but as part of the province’s opioid crisis strategy (Government of Ontario, 2016). Similarly, widely disseminated clinical practice guidelines for opioid prescribing for chronic pain were released in Canada in 2017. One of their 10 recommendations stated, “For patients with chronic noncancer pain who are using opioids and experiencing serious challenges in tapering, we recommend a formal multidisciplinary program” (Busse et al., 2017). By contrast, Canada and few other comparable jurisdictions, have yet to develop clinical practice guidelines or strong health system strategies for chronic pain management. Taken together, these suggest a trend of returning interest in MDC—not MDC for the sake of improving pain management, but MDC for the sake of reducing harms from opioids. These are clearly distinct justifications that may have implications for the design, delivery, evaluation and long-term support of programs.

To help clarify the guidance around what constitutes MDC for opioid dose reduction, how these programs are constituted and how they might be expected to work, we conducted a realist systematic review of MDC for opioid dose reduction (Sud et al., 2020). Through our multi-pronged, systematic search strategy, we identified 95 English-language evaluations of MDC programs for people living with chronic noncancer pain that reported opioid dose outcomes. These studies were from 9 countries, though the majority were from the US. They spanned five decades from the 1970s through to the 2010s.

**Fig. 1:**
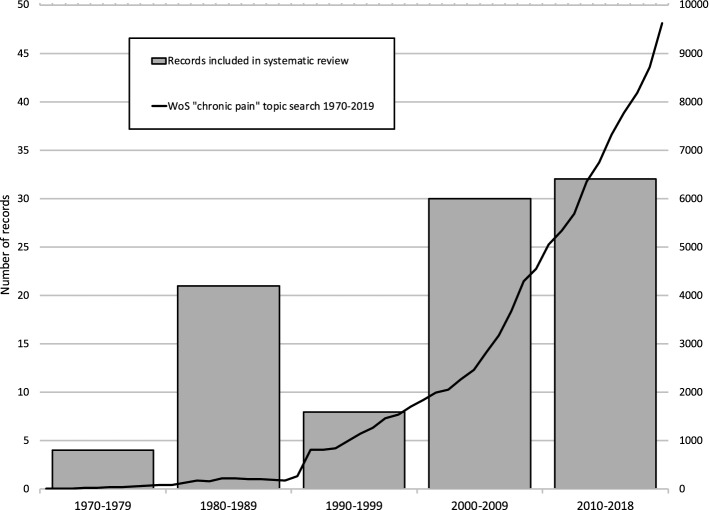
Included studies by decade (from Sud et al., 2020)

Systematic reviews aim to collect the entirety of a defined set of literature to answer a specific research question. As such, they provide an opportunity to also trace the dynamics of scientific evidence production and thought for that particular subject or field. In the annual distribution of the publication dates for the included studies in our realist review, we identified a peculiar trend. While there was a steady increase in the number of publications between the 1970s and 1980s, there was a notable decline in the 1990s with less than 10 studies published in this decade. This was subsequently followed by a sharp rise in the number of included publications in the 2000s and 2010s. This is despite steady increases in scientific publications generally and chronic pain related publications specifically (Fig. 1).

The objective of this study is to determine if these temporal trends represent a change in the scientific perception and utilization of MDC with transitions in the scientific literature being driven by the developments of the opioid crisis. We hypothesized that the early studies reflected a biopsychosocial approach to chronic pain care, that the dip in the 1990s was due to a biomedicalization of chronic pain care and thus displacement of attention away from MDC towards other therapies (chiefly opioid analgesics), and that the return of interest and scientific productivity through the 2000s represents an attempt to mitigate harms from the growing opioid crisis rather than a return of interest in biopsychosocial chronic pain care itself. We further hypothesized that the changes in these specific periods would correlate to the rising opioid-related harms in the US where a majority of the literature was published.

## Methods

We conducted a quantitative bibliometric study using both time series and content analysis. We had two orders of data. The first order data were the 95 studies collected in our systematic realist review (Sud et al., 2020). The second order data were the set of publications that cited any one of these 95 studies. We used the second order data to examine for the replication of any trends in the first order data and then also its subsequent scientific effects.

## Data sources

The first order set was collected through a multi-pronged search strategy identifying evaluations of MDC programs that reported on opioid dose outcomes. Between May and June 2018, we searched multiple bibliographic databases (MEDLINE, PsychINFO, AMED, CINAHL Plus and Cochrane Library). This was supplemented with a targeted search of EMBASE (conference proceedings and meeting abstracts), Cochrane CENTRAL Trials database as well as ClinicalTrials.gov (clinical trial data), Proquest Dissertations and Theses (Dissertations), and four null and negative results journals. We also conducted a grey literature search and a hand-search of references of included and relevant articles. We restricted our search to English-only publications.

In July 2020, we extracted the number of citations and related metadata for first order studies from Web of Science Core Collection (WoS), Scopus and Google Scholar. This constituted our second order data set. We elected to use WoS as our primary database as this included a high percentage of publications from our first order set and also had the most comprehensive bibliometric metadata to conduct our analysis.

## Outcome measures

### Content analysis

We conducted a content analysis of our first order data to determine two characteristics of each publication. First, as part of our original synthesis, we extracted data about the requirement for opioid tapering as part of participation in the program. We used this outcome as a measure of the program’s attitude towards the appropriateness of opioid prescribing for chronic pain management. Two reviewers independently examined the program descriptions and determined whether opioid tapering was a required, suggested, or not required component of program participation. We noted that close to 60% of programs required opioid tapering, 10% suggested opioid tapering and the remainder either had no protocol, did not report on the requirement or were unclear. For the purposes of this study, we coded this as a binary variable with required or suggested opioid tapering versus no requirement or unclear requirements.

Second, we reviewed the first order data set to determine whether each publication referenced population-level opioid-related harms or the opioid crisis as a motivation for the existence of the program being described or justification for the evaluation being reported. We coded this variable categorically as: (1) agenda-setting (Thelwall, 2019); (2) any reference (but not agenda-setting); or, (3) no reference to opioid-related harms, to an opioid crisis or to an opioid epidemic. This content analysis was done independently and in duplicate after an initial calibration exercise with 10 randomly selected publications from the data set. Any disagreements were resolved by consensus or discussion with a third reviewer.

### Bibliometric outcomes

Next, we extracted bibliometric metadata for both the first order and second order data sets. The variables collected, based on WoS definitions, included: authors, year of publication, country, research area, document type and funding. In cases where two research areas were identified, we included both. When research areas for specific records were not identified by WoS, the record was examined independently by two authors and research areas were imputed by comparing to similar records that had research areas assigned by WoS. We managed disagreements on the imputation through discussion and made a consensus decision. For funding, we examined the WoS output and assigned each record categorically as either commercial funding, no commercial funding or not reported. Commercial funding included any private, for-profit funding source, including when there was a combination of private, for-profit and public funding.

### Opioid-related harms

Finally, we used data extracted from the CDC WONDER online database and reports from the US Department of Health and Human Services to collect drug and opioid overdose mortality data (CDC, 2000; Hedegaard et al., 2020). We noted a significant change in definitions for drug mortality between 1998 and 1999, so collected two distinct data sets (1975–1998 and 1999–2019) that could not be combined for the period of interest for this study (1975–2019).

## Data analysis

Proportions between categories were compared using Chi-squared or Fisher Exact tests. The Chow test was used to test for the presence of structural break points in time series data (Chow, 1960). Segmented regression of interrupted time series analysis with AR(2) residuals were used to examine the sudden and gradual change in the proportions of publications of interests at given time points. We used cross-correlation functions to assess correlation and possible lag delay between two times series (Wagner, 2002). The statistical software SAS 9.4 was used for statistical analysis. All tests were two sided and *p* < 0.05 was considered statistically significant.

## Results

We initially examined three widely used and comprehensive bibliometric databases to determine the most appropriate database for our study. (Table 1)

**Table 1:** Bibliometric database indexing and citations

Database	Number of first order studies indexed (%)	Total number of citations
Web of Science	88 (92.6)	3398
Scopus	89 (93.7)	3735
Google Scholar	95 (100)	6376

## First order data

### Time series analysis

**Fig. 2:**
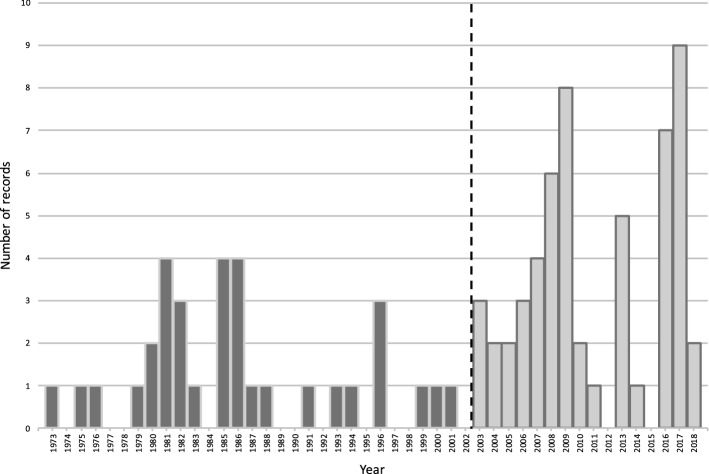
First order time series (n = 88), 1973–2018

We plotted the indexed first order records (n = 88) by publication year to construct our first order time series. Visual inspection suggested a different pattern compared to the plot by decade (Fig. 2). There was intermittent activity through the 1970s and 1990s, followed by increases from the mid-2000s onwards. We used the Chow test with log(count + 1) as the outcome to determine a break point in the time series between 1995 and 2007. The only significant change during this period was in 2003 (*p* = 0.0404).

To confirm this breakpoint, we fitted a segmented regression of the form $y_{t} = b_{0} + b_{1} \times t{\text{ime}}_{t} + b_{2} \times {\text{intervention}} + b_{3} \times {\text{time}} - {\text{after}}_{t} + e_{t}$ with $\log \left( {{\text{count}} + 1} \right)$ (Wagner et al., 2002). This analysis identified no trend before 2003, while there was a positive and significant upward trend after 2003 (*p* = 0.0318). The diagnostic plots showed adequate model fit. Finally, we fit a step function which showed that the average of publications before 2003 and from 2003 and afterwards were statistically different (*p* < 0.0001).

### Categorical analysis

Using this time series analysis, we categorized our first order data into the 1973–2002 and 2003–2018 periods and compared across our bibliometric metadata (Table 2). Of the 88 indexed records, the majority were published in the US (73.9%). The most common research areas were neuroscience/neurology (“NEURO”, 29.2%), anaesthesiology (“ANESTH”, 26.9%), general internal medicine (“GIM”, 16.2%), psychiatry and psychology (“PSYCH”, 10.0%) and rehabilitation (“REHAB”, 3.8%). The majority of the publications were of programs that required opioid tapering as part of participation in the program (55.7%). At the same time, 43.2% of the articles recognized opioid-related harms as agenda setting.

**Table 2:** First order Country, Research area, Opioid harms, Opioid tapering by time period (1973–2002 and 2003–2018)

Variable	Outcome	Total (%)	Number (%) 1973–2002	Number (%) 2003–2018	*p* value
Country	United States	65 (73.9)	26 (78.8)	39 (70.9)	0.6826
	United Kingdom	5 (6.7)	3 (9.1)	2 (3.6)	
	Australia	5 (5.7)	1 (3.0)	4 (7.3)	
	Canada	5 (4.6)	1 (3.0)	3 (5.4)	
	Denmark	3 (3.4)	1 (3.0)	2 (3.6)	
	Sweden	1 (1.1)	1 (3.0)	0 (0.0)	
	Finland	1 (1.1)	0 (0.0)	1 (1.8)	
	Germany	3 (3.4)	0 (0.0)	3 (5.4)	
	Japan	1 (1.1)	0 (0.0)	1 (1.8)	
	TOTAL	88 (100.0)	33 (37.5)	55 (62.5)	
Research area	NEURO	38 (29.2)	14 (28.6)	24 (29.6)	0.0265
	ANESTH	36 (26.9)	**11 (22.4)**	**24 (29.6) ↑**	
	GIM	21 (16.2)	**4 (8.2)**	**17 (21.0) ↑**	
	PSYCH	13 (10.0)	**10 (20.4)**	**4 (3.7) ↓**	
	REHAB	5 (3.8)	2 (4.0)	3 (3.7)	
	OTHER	18 (13.8)	8 (16.3)	10 (12.3)	
Reference to opioid harms	Agenda-setting	38 (43.2)	**9 (27.3)**	**29 (52.7) ↑**	0.0125
	Non-agenda-setting	12 (13.6)	**3 (9.1)**	**9 (16.4) ↑**	
	None	38 (43.2)	**21 (63.6)**	**17 (30.9) ↓**	
Opioid tapering	Required	49 (55.7)	16 (48.5)	33 (60.0)	0.3761
	Not required	39 (44.3)	17 (51.5)	22 (40.0)	

Bolding and arrows indicate trends of relevance

*NEURO* neuroscience/neurology, *ANESTH* anesthesia, *GIM* general internal medicine, *PSYCH* psychiatry/psychology, *REHAB* rehabilitation

There were no statistically significant changes between the two time periods in terms of the country of origin or for the requirement of opioid tapering. The research areas demonstrate significant changes with increases in ANESTH and GIM categories, mostly at the expense of PSYCH. The contributions of the NEURO, REHAB and OTHER fields stayed consistent. Likewise, there was a near doubling of the proportion of studies with agenda-setting or any reference to population-level opioid-related harms between the first and second periods.

## Second order data

### Time series analysis

**Fig. 3:**
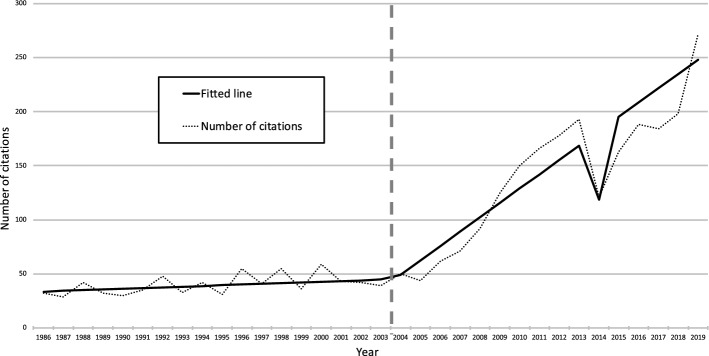
Second order time series (1986–2019), segmented regression

Using the same procedure as the first order time series, segmented regression of the second order citation data demonstrated a clear trend change from 2004 (Fig. 3). For the purposes of this segmented regression, we excluded the pre-1986 period which visually demonstrated a distinct dynamic with a sustained peak of activity before the nearly 20 years of lower-level activity, which accords with historical accounts of the rise of pain MDC (Meldrum, 2003; Schatman, 2010). We further considered 2014 as an anomaly and created a dummy variable (1 when year = 2014, and 0 otherwise) to account for this. Thus, our segmented regression examined the time series for the period of 1986–2019. The period between 1986 and 2003 showed no trend (slope = 0.65, *p* = 0.5050), while the period from 2004 onwards showed a clear increasing trend of 12.6 citations annually (*p* < 0.0001), suggesting a structural breakpoint at 2004 and identifying 1986–2003 as one period and 2004–2019 as second period. This inflection point was confirmed with negative binomial segmented regression, with no trend from 1986 to 2003 (*p* = 0.360) and slope of 14.3 (95% CI 10.5, 18.1) from 2004 to 2019 (Fig. 3). No other structural breakpoints in the time series were identified. Overall, this procedure identified three periods of 1975–1985, 1986–2003, and 2004–2019, each with distinct time-based dynamics.

### Categorial analysis

Using this time series based categorization, we compared our bibliometric metadata across each period (Table 3).

**Table 3:** Second order Country, Research area, Document type, Funding, Opioid harms and Opioid tapering by time period (1975–1985, 1986–2003 and 2004–2019)

Variable	Outcome	Total (%)	Number (%) 1975–1985	Number (%) 1986–2003	Number (%) 2004–2019	*p*
Country	United States	1960 (57.7)	**244 (75.3)**	**360 (49.7) ↓**	1356 (57.7)	< 0.0001
	Canada	216 (6.4)	24 (7.4)	50 (6.9)	142 (6.0)	
	England	211 (6.2)	6 (1.8)	91 (12.6)	114 (4.8)	
	Germany	181 (5.3)	4 (1.2)	57 (7.8)	120 (5.1)	
	Australia	170 (5.0)	7 (2.2)	40 (5.5)	123 (5.2)	
	Sweden	96 (2.8)	26 (8.0)	32 (4.4)	38 (1.6)	
	Netherlands	87 (2.6)	2 (0.6)	36 (5.0)	49 (2.0)	
	Other	477 (14.0)	11 (3.4)	58 (8.0)	408 (17.4)	
	TOTAL	3398 (100.0)	324 (9.5)	724 (21.3)	2350 (69.2)	
Research area	NEURO	1224 (23.6)	**82 (17.2)**	**308 (26.6) ↑**	834 (23.6)	< 0.0001
	ANESTH	1003 (19.4)	**76 (15.9)**	**239 (20.6) ↑**	688 (19.4)	
	GIM	638 (12.3)	24 (5.0)	**87 (7.5)**	**527 (14.9) ↑**	
	PSYCH	634 (12.2)	**170 (35.6)**	**183 (15.8) ↓**	**281 (8.0) ↓**	
	REHAB	303 (5.9)	44 (9.2)	**94 (8.1)**	**165 (4.7) ↓**	
	OTHER	1366 (26.4)	82 (17.2)	246 (21.2)	1038 (29.4)	
Document type	Article	2518 (74.1)	238 (73.5)	571 (78.9)	1709 (72.7)	< 0.0001
	Review	694 (20.4)	69 (21.3)	121 (16.7)	504 (21.4)	
	Editorial	149 (4.4)	**4 (1.2)**	**22 (3.0) ↑**	**123 (5.2) ↑**	
	Other	37 (1.1)	13 (4.0)	10 (1.4)	14 (0.6)	
Funding	Any industry	165 (4.9)	0 (0.0)	0 (0.0)	165 (7.0)	< 0.0001
	No industry	1020 (30.0)	19 (5.9)	**66 (9.1)**	**935 (39.8) ↑**	
	Not reported	2213 (65.1)	305 (94.1)	**658 (90.9)**	**1250 (53.2) ↓**	
Reference to opioid harms	Agenda-setting	997 (29.3)	**17 (5.2)**	**109 (15.0) ↑**	**871 (37.1) ↑**	< 0.0001
	Mention only	335 (9.9)	11 (3.4)	**10 (1.4)**	**314 (13.4) ↑**	
	No mention	2066 (60.8)	296 (91.4)	**605 (83.6)**	**1165 (49.6) ↓**	
Opioid tapering	Required	2151 (63.3)	184 (56.8)	**392 (54.1)**	**1575 (67.0) ↑**	< 0.0001
	Not required	1247 (36.7)	140 (43.2)	**332 (45.9)**	**775 (33.0) ↓**	

Bolding and arrows indicate trends of relevance

*NEURO* neuroscience/neurology, *ANESTH* anesthesia, *GIM* general internal medicine, *PSYCH* psychiatry/psychology, *REHAB* rehabilitation

There were 3398 citations of the 88 first order studies indexed in WoS. The median number of citations per first order study was 22.5. Twenty-four (27.3%) studies were cited 10 or less times, 43 (48.9%) were cited 11–50 times, 14 (15.9%) were cited 51–100 times and 7 (8.0%) were cited more than 100 times.

The most frequent country of origin was again the US and the most common research areas were NEURO and ANESTH (Table 3). Articles were the most frequent publication type (74.3%) and the majority (65.1%) did not report funding. Finally, 63.0% of these studies had cited first order records that required opioid tapering and 60.8% cited first order records that did not reference population-level opioid-related harms.

Records in the most recent period (2004–2019) originated from a more diverse international research community, though the contribution from the US was consistently near or above 50%. Articles originating from Sweden and Germany were less likely to reference articles from the first order set that required opioid tapering. Likewise, articles from Sweden (11.5%) and the Netherlands (5.8%) were much less likely to reference articles from the first order set that made agenda-setting references to the opioid crisis (other countries were consistently > 20%). Studies from these two countries also were more likely to be published in REHAB and PSYCH fields and had lower proportions of editorial and review materials as compared to primary research articles.

**Fig. 4:**
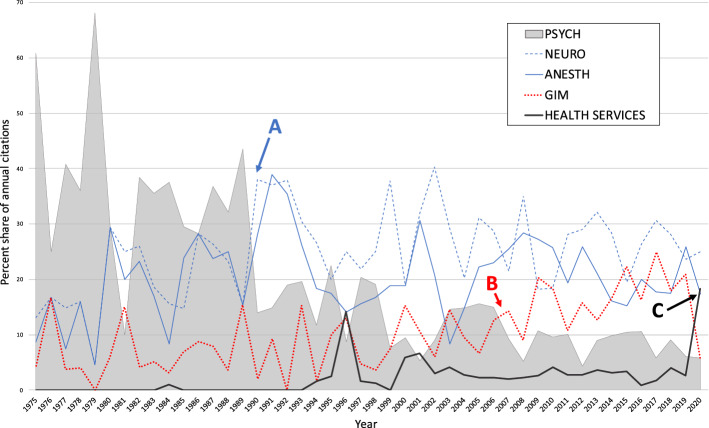
Percent share of annual citations by Research area (second order data set). **A** NEURO and ANESTH surpass PSYCH, **B** GIM surpasses PSYCH, **C** large spike in HEALTH SERVICES. Abbreviations: NEURO = neuroscience/neurology, ANESTH = anesthesia, GIM = general internal medicine, PSYCH = psychiatry/psychology, REHAB = rehabilitation

The change in the research area was similar to that seen in the first order data set. Both NEURO and ANESTH showed early increases and their contributions were sustained into the later period. Both NEURO and ANESTH surpass the percentage contributions of PSYCH by 1990 (point A, Fig. 4). There was a substantial and significant increase in GIM records only in the later period (8.5 to 16.1%) which mostly came at the expense of PSYCH (and also REHAB) records (point B, Fig. 4). There is a very late spike in the HEALTH SERVICES area in 2020, representing 18.3% of the total compared to under 5% for most of the previous two decades (point C, Fig. 4). No publications in this data set, including specifically from 2020, were related to SARS-CoV-2 or COVID-19.

Throughout the study period, ANESTH, NEURO and GIM records were much more likely (*p* < 0.0001) to reference articles that made agenda-setting reference to the opioid crisis (40.9%, 33.0%, 31.6% respectively) than were REHAB (12.9%) or PSYCH (16.1%). We noted that ANESTH, GIM and NEURO records were more likely to be reviews and editorials than were REHAB and PSYCH articles (*p* < 0.0001, not shown). Besides a consistent increase in editorial materials, which made up a small proportion of the records, we did not note any other significant trends over time with respect to document types. Research articles dominated each time period at > 72% throughout.

There was an overall increase in reporting of funding in the later period (7.0% compared to 0.0% in the two earlier periods), though 53.2% of records in the later period still did not make any funding disclosures. Throughout the study period, records which referenced articles for which the opioid crisis was agenda-setting showed the highest proportion of funding from industry (6.6% versus 3.3% and 2.4%, for any reference and no reference, respectively). The proportion of records citing first order studies that required opioid tapering showed no changes between the two early periods and then increased significantly from 54.1 to 67.0% into the later period. Likewise, there were significant increases in the number of citations of studies for which there were agenda-setting references to population-level opioid-related harms (5.2% to 15.0% to 37.0% from the earliest to latest periods). The proportion of records that referenced primary studies with any reference to population level opioid-related harms also increased from 1.4 to 13.4% between the last two periods.

### Comparing the first order and second order data

**Fig. 5:**
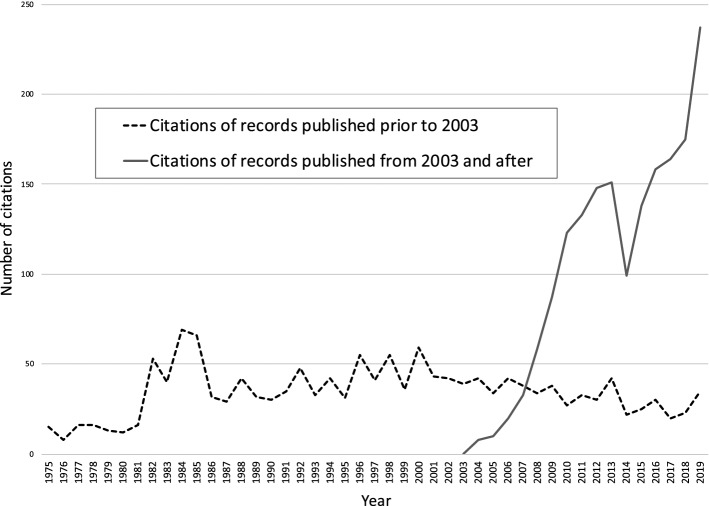
Decomposed second order time series—citations of records published prior to and post-2003

To compare the first order and second order data sets, we decomposed the second order time series based on whether the studies cited the 1973–2002 first order studies or the 2003–2018 first order studies (dotted and solid lines, respectively, in Fig. 5).

**Fig. 6:**
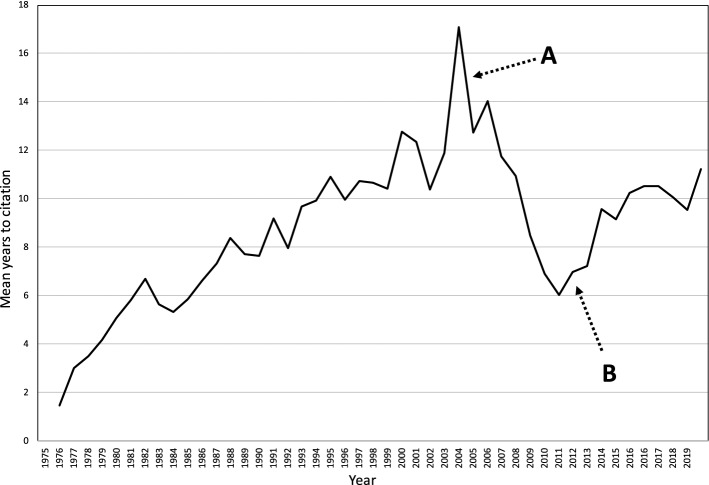
Mean years to citation, second order data set. **A** Sharp decrease in mean years to citation, **B** recovery in mean years to citation

It is evident in this decomposed time series that the total number of studies citing the first order 1973–2002 set stays consistent. Namely, the significant change in 2004 in the second order data set is entirely attributable to first order studies published after 2003. To confirm this finding, we first compared the slopes of the pre-2003 citations from the 1985–2003 period (dotted line) to the 2004–2019 period (also dotted line) and found that both showed no significant trend (*p* = 0.4717). Second, we calculated the mean number of years to citation by subtracting the year of publication of the first order record from that of the second order record and dividing by the total number of second order records per year. We then plotted this as a time series (Fig. 6).

This plot demonstrates a clear and sustained increase in mean years to citation between 1975 and 2004, as would be expected when citing an established data set. There is a discontinuity starting in 2005 with a sharp decrease in the mean years to citation (point A, Fig. 6). This trend begins to recover only after 2011 (point B, Fig. 6).

### Comparing the second order time series to opioid-related mortality data

**Fig. 7:**
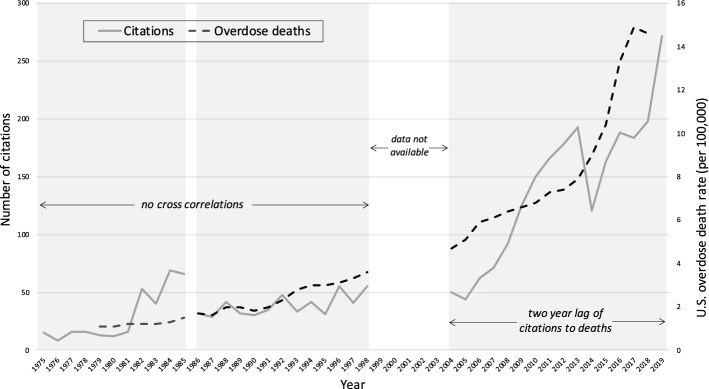
Second order citation time series with US opioid-related mortality time series

Cross-correlation between the second order time series and the time series for opioid-related deaths in the US demonstrated specific patterns across the three time periods (Fig. 7). For both the first period of 1979–1985 and second period of 1986–1998, both citation slopes and opioid overdose mortality are increasing, however there was no significant cross-correlation (*p* > 0.05). For the 2004–2019 period, we see both time series increasing dramatically. The cross-correlation function shows a moderate, two-year lag of the citation trend compared to the death rate (*p* = 0.0385).

## Discussion

### Summary of results

We identified three distinct periods relating to MDC for opioid dose reduction with some evidence that there may be a current transition into a fourth period (Table 4). The first period represents an early consolidation of the effectiveness literature examining MDC programs for chronic pain management. This period was dominated by the PSYCH and REHAB fields and represented a biopsychosocial approach to chronic pain management in which opioids were used but typically at low doses and rarely as monotherapy. This is affirmed by historical accounts of MDC, which were often pioneered by anaesthetists but then gained wider success and traction when combined with psychological and behavioural approaches (Bonica, 1990). A first wave of biomedicalization began in the late 1980s and a second period was thus established by 1990 through the ANESTH and NEURO fields. This coincides with the movement for wider assessment and treatment of pain, such as the “pain as a 5th vital sign” movement, driven by the American Pain Society starting in the early 1990s, including with funding and support from the pharmaceutical industry (Rummans et al., 2018). The beginnings of this period precede what many point to as the origin of the contemporary opioid epidemic in North America, namely the release and marketing of Oxycontin® by Purdue Pharma in 1996, which was associated with a steep rise in opioid consumption in high income North America (Dhalla et al., 2009).

**Table 4:**
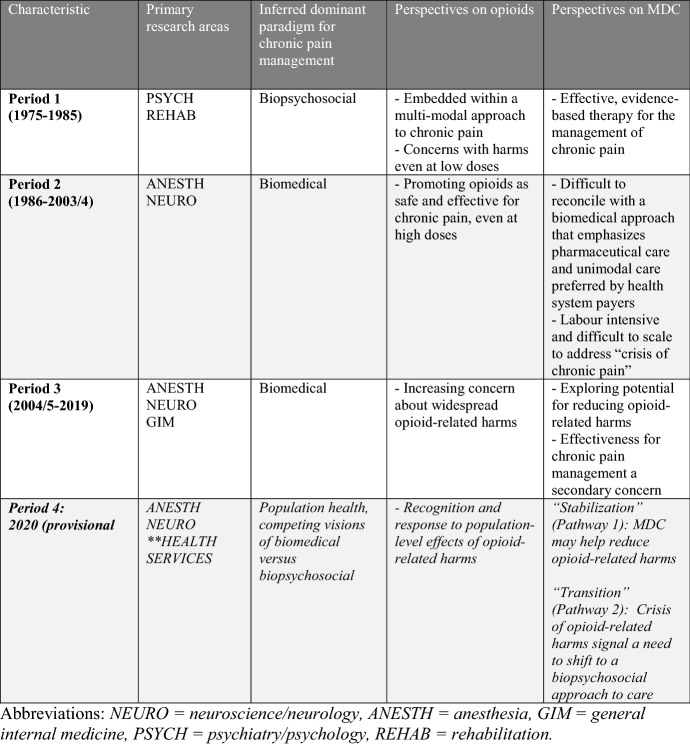
Summary of three periods (1975–2019) with provisional fourth period (2020)—Research areas, Chronic pain paradigm, Perspectives on opioids and Perspectives on MDC

This study strongly suggests that 2003–4 was an important transition phase in the MDC for opioid dose reduction literature. In the third period, we see a second wave of biomedicalization particularly from the GIM literature with increasing consternation about opioid-related harms (as represented by increasing citations of studies that made agenda-setting references to opioid-related harms) and specifically increasing references to MDC programs that required opioid tapering. This new wave is characterized by a sort of scientific “memory lapse” with a stark failure to cite and therefore recognize the more biopsychosocially-oriented first order publications from before 2003. By 2011, the mean time to citation approximates what it was a quarter of a century earlier in 1986–it is as if this at least 30 year-old field began to start afresh after 2004.

These timelines coincide with the North American opioid crisis more generally. Sales of OxyContin® had reached $1.1 billion by 2000 and by 2004 this same drug had become a leading drug of extramedical use in the US (Van Zee, 2009). Likewise in 2004, Purdue Pharma faced its first lawsuit regarding OxyContin®, by the state of West Virginia, over excessive prescription costs. It was just a few years later in 2006 that the US Centres for Disease Control first started compiling national statistics on opioid prescribing. These timelines are also reflected in an analysis of national Canadian media coverage of opioids which began to rise only around 2004 suggesting this timeframe as the beginnings of wider popular awareness of opioid-related harms (Webster et al., 2020). That this third period is responding to the opioid crisis is further confirmed by the cross-correlation which showed that the citation trends significantly lagged the US opioid death trends by two years.

We also identified distinct international trends with some northern European countries, such as Sweden and the Netherlands, demonstrating a continued, distinct biopsychosocial orientation. Opioid consumption rates have risen through much of Europe during the period under study here, but not nearly at the scale seen in high income North America and specifically without the stark inflection point in the mid-1990s (Pain & Policy Studies Group, 2016). Perhaps a continued orientation and commitment to a biopsychosocial approach to chronic pain management in parts of Europe has been somewhat protective against increasing opioid prescribing, consumption and widespread harms.

## Implications

### The sociotechnical embedding and transitions of health technologies

First, this study suggests that there is benefit in understanding the cognitive framing of opioid analgesic use in explaining its health and social effects. Within a biopsychosocial framing, opioids are embedded within multiple modes of chronic pain care including non-pharmacological treatments, which help to constrain their use (Flor et al., 1992). Within a biomedical frame which prioritizes pharmaceutical interventions and unimodal care, opioid analgesics can be used much more widely and without the protective and constraining forces of other forms of effective therapy. As others have argued, such a “sociotechnical” framing of health technologies such as pharmaceuticals can be a fruitful line of inquiry in understanding their utilization and effects, which are mediated by not just the technical or pharmacological properties of drugs but also by their social embedding (Whyte et al., 2002). Schatman (2011) has also argued that the “demise” of chronic pain MDC against the rise of opioids was driven by a shift from (medical) professional values of suffering and improving patient health to particular economic values of cost-cutting and profit-making, the demonstrated cost-effectiveness of pain MDC notwithstanding. This emphasizes that the normative embedding of health technologies need be considered alongside the cognitive embedding.

While the data from 2020 are preliminary, this analysis suggests that the scientific literature and thus sociotechnical embedding of opioids may be moving to a new phase focused on population health perspectives on the opioid crisis. Drawing from frameworks for sociotechnical transitions (Geels & Schot, 2007), we see two possibilities for this population level response as it pertains to MDC and opioid use. The first would be a *stabilization* of the biomedical perspective. This would be characterized by attempts to reduce opioid-related harms for example by large-scale opioid deprescribing efforts or attempts to develop “non-addictive” opioid analgesics, both of which have been important elements of the contemporary response to the opioid crisis. These efforts do not specifically challenge the unfettered embedding of pharmaceuticals in contemporary medical care. Alternatively, the population health and health services literature, in grappling with the increasing consequences of the opioid crisis, may justify a *transition* to a more holistic, biopsychosocial approach to chronic pain management. This would imply a different, constrained embedding of pharmaceuticals and a reorientation of care, and care reimbursement, away from unimodal approaches.

That these two possibilities are alive in the contemporary landscape is suggested by the various priorities of the multibillion dollar Helping to End Addiction Long-term (HEAL) initiative of the US National Institutes of Health developed in response to the opioid crisis. The priorities contain elements of these competing visions such as substantial funding for novel pharmaceuticals but also for alternative, non-pharmaceutical and multi-modal treatments for the chronic pain (Collins et al., 2018; “The Helping to End Addiction Long-term Initiative”, 2019). Using the insights developed from this study, we expect that further bibliometric analysis of this emerging health services literature would be able to adjudicate between these two kinds of sociotechnical transition possibilities in two specific ways. First, one could examine for the time to citation in the health services field. A shorter time to citations with citations focused on the post-2004 period would suggest a biomedical *stabilization*. A longer time with inclusion of citations from the pre-2003 period would suggest elements of a *transition* back to a biopsychosocial approach. Second, a stabilization trajectory would be suggested by a higher proportion of citations of MDC programs with required opioid tapering protocols, reinforcing this phenomenon of MDC as a way to focus on reducing opioid prescribing rather than focus on appropriate management of chronic pain.

### Bibliometrics as both reflective and generative of real-world phenomena

This analysis suggests a strong, two-way correlation between the scientific literature and a defining health and social issue of our time. We have demonstrated that in the 2004–2019 period, the scientific literature lagged opioid deaths rates by two years, suggesting that the scientific community is reflecting on and responding to this widespread health and social phenomenon. This is reinforced by the increasing proportions of editorial materials in this corpus as compared to primary empirical studies. However, there are suggestions here as well that the scientific literature may have been *generative* of this health and social phenomenon. While much of the attention on the iatrogenic origins of the opioid crisis has focused on the release and marketing of Oxycontin® in 1996, this analysis suggests that a wave of biomedicalization preceded this time point by nearly a decade. Cognitive and normative changes driven by the scientific literature may have set the stage for the widespread consumption and use of opioids. An increasing biomedical approach to chronic pain management paved the road for increased pharmaceutical utilization, displacing the multimodal and multidisciplinary approach associated with biopsychosocial conceptions of chronic pain. Indeed, other bibliometric analyses of specific publications have aimed to identify this *generative* role of the scientific literature, which provided clinicians the scientific justification and biomedical license to widely prescribe high-dose opioids for the management of pain (Leung et al., 2017).

This bidirectional relationship between the scientific literature and real-world phenomena points to an important utility of bibliometric analyses of systematic reviews, and that specifically bibliometrics can have important roles beyond measures of scientific productivity and relationality. Co-citation networks of representative samples of research domains have provided important insights into the development, dynamics and character of various defined fields (Chen, 2017). To our knowledge, this study is the first application of time series analysis to study the (unsteady) temporal dynamics of a specific scientific field. Considering the accelerating pace with which systematic reviews are being conducted globally (Ioannidis, 2016) and the significant resources required to conduct such reviews, there is a substantial opportunity for additional knowledge generation both about science but also about the real-world phenomena that scientific fields are studying, by using bibliometric analyses as a complement to systematic reviews.

## Limitations

The major limitation of this study is that the original systematic review included English language-only records. This may have biased our findings towards a particular conceptualization of MDC for opioid dose reduction and also maintained a particular focus on US publications and phenomena. Besides the English-language focus, this US-centredness may have been expected given the relative scientific productivity of the US (Monroy & Diaz, 2018) as well as the increased utilization of opioid analgesics and then subsequent prescription opioid-related harms in that country. Importantly, however, this US focus also facilitated a meaningful comparison to national-level opioid death data that may not have been possible with a literature set that was more diverse in national origin.

There were seven (7.4%) first order records not indexed in WoS. This sample was too small to detect any systematic differences between indexed and non-indexed records. Importantly, we did not detect any important difference in the time series dynamics between the full first order set and slightly smaller WOS-indexed set. This number was comparable to the number of non-indexed records in Scopus (6) and four of the non-indexed records were common. To determine whether there were any systematic errors introduced by the choice of bibliometric database, a similar analysis could be conducted using Scopus and the results could be compared.

In the second order time series, 2014 represented a clear anomaly and was statistically treated as such. We could not identify any real-world reason why there would be such a substantial drop off in productivity for this single year and thus felt justified in this approach to analysis. However, if this did represent a reflection of some real phenomenon that our analysis should have accounted for, then this could change how the dynamics of the various time periods compare.

## Conclusion

To our knowledge, this study represents the first example of using time series based bibliometrics to analyse a body of scientific literature from a systematic review. We identified three distinct periods of scientific activity with progressive waves of biomedicalization throughout the 44-year study period. Specifically, we found that 2003/2004 was an important inflection point in the scientific literature relating to multidisciplinary care and opioid use. Likewise, we identified important cognitive and normative shifts in the scientific literature in the decade prior to the release of OxyContin®, which has been popularly recognized as the origin of the contemporary opioid crisis.

These scientific dynamics correlate, likely bidirectionally, with the real-world phenomenon that they study, suggesting not only a reflective but also generative role of science in the contemporary opioid crisis. We identified an important scientometric phenomenon which we have dubbed as a scientific “memory lapse” with a distinct failure to cite older, though relevant, literature during a specific bibliometric and social transition point. We propose that this metric of time to citation can be used predictively to interpret emerging contemporary dynamics in the literature, and thus about likely directions of scientific, clinical and policy responses to the opioid crisis and other phenomena of future interest. Given the contemporary proliferation of systematic reviews in many scientific fields, and given the substantial resources invested in conducting these systematic reviews, associated bibliometric analyses offer important means for generating useful knowledge about the scientific field and potentially the phenomenon of interest to the field.

## Data Availability

The data that support the findings of this study are available from the corresponding author, upon reasonable request.
